# Predictors of Depression and Insomnia in Community-Dwelling Elderly People: A Cross-Sectional Evidence of Their Bidirectional Relationship

**DOI:** 10.7759/cureus.13965

**Published:** 2021-03-18

**Authors:** Konstantinos Tsaras, Maria Tsiantoula, Ioanna V Papathanasiou, Dimitrios Papagiannis, Maria Chatzi, Evangelos C Fradelos

**Affiliations:** 1 Department of Nursing, University of Thessaly, Larissa, GRC; 2 Department of Welfare, Municipality of Larissa, Larissa, GRC; 3 Community Nursing Lab, Faculty of Nursing, University of Thessaly, Larissa, GRC; 4 Department of Infection, University Hospital of Larissa, Larissa, GRC

**Keywords:** depression, insomnia, elderly people, older adults, geriatric depression scale, athens insomnia scale

## Abstract

Objective

The aim of this study was to examine associated factors of depression and insomnia in community-dwelling elderly people in order to identify independent predictors.

Materials and methods

A cross-sectional study was conducted among 250 older people aged 60 years and over living in an urban area. A stratified random sampling method was used for recruiting samples from five Open Care Centers for Elderly People of the Municipality of Larissa, Greece. Data were obtained through a questionnaire that included demographic, socioeconomic, and health-related characteristics, 15-item Geriatric Depression Scale (GDS-15), and Athens Insomnia Scale (AIS). Simple and multiple logistic regression analysis was performed.

Results

The prevalence of depression and insomnia was 28.4% (95% CI: 22.9-33.9) and 39.2% (95% CI: 33.0-45.4), respectively. Our findings showed that the overall GDS-15 score was positively related to the overall AIS score (*r* = 0.405; *p* < 0.001). The best-fit regression analysis demonstrated four significant predictors (marital status, monthly income, body weight status, and insomnia) explaining 31.6% of the variance in depression risk. Moreover, community-dwelling elderly Greek people with insomnia symptoms were females, had a lower monthly income, and more likely to suffer from chronic diseases and depression.

Conclusion

These findings point to the importance of recognizing risk factors for both depression and insomnia in attempting to apply preventive interventions in the elderly and optimize their quality of life.

## Introduction

According to the United Nations data, there were 727 million persons aged 65 years or above in the world in 2020. The overall reduction in fertility causes the proportion of older people to increase over time. Globally the number of elderly people is expected to more than double by 2050 [1]. A high proportion of elderly people are reported to have “poor mental health”.

Depression is the most common mental health disorder in the elderly population and is associated with functional, cognitive, physical, and social impairments [2]. A recent meta-analysis of 120 studies, which were conducted in South Asian countries, revealed that the overall pooled prevalence of depression among the elderly was 42.0% [3]. A previous meta-analysis of 25 studies from Western countries (Europe and North America) found a prevalence of depressive symptoms among older adults living in the community at 19.5% [4]. Epidemiological data from eight large aging cohort studies from 18 countries around the world estimated the overall prevalence of geriatric depression at 29.6% (SD = 45.6), which ranged from 17.1% (Denmark) to 63.7% (Chile) [5]. Differences in estimates of the prevalence of depression among elderly populations may be related to the use of different methodologies, depression screening tools, and characteristics of the samples, and probably due to different sociocultural contexts between countries.

The literature has been identified demographic determinants (e.g., older age, female gender, unmarried status), lower socioeconomic status (e.g., lower educational level, financial hardship), poor social support, existence of psychosocial stressors, cognitive impairment, general health-related factors (e.g., obesity, smoking, alcohol use), and comorbid physical illness as common risk factors of late life depression [2]. Similarly, in a recent cross-sectional study among 378 community-dwelling elderly people living in urban and semi-urban areas in southwestern Greece, depressive symptoms were associated with female gender, being unmarried, low educational level and household income, sleep disturbance, and comorbidity [6].

Insomnia is the most common and widespread sleep disorder among people aged 60 years or older and a frequently overlooked risk factor for late life depression. It is characterized by the subjective complaint of difficulty falling or maintaining sleep, or non-restorative sleep, causing symptoms of fatigue or malaise, difficulty in concentration or memory impairment, and mood disturbances or irritability during the daytime [7]. The prevalence of insomnia symptoms in the elderly population is estimated to range from 30% to 48% and the prevalence of insomnia disorder from 12% to 20% [8]. The high frequency of insomnia in the aging population probably indicates the critical role of neuro-degeneration in the pathophysiology of sleep disorder.

Several correlates of insomnia have been demonstrated in the elderly, such as sociodemographic determinants (e.g., older age, female gender, lower levels of education and income, current working status), stressful life events, cognitive function and dementia, medical (e.g., respiratory symptoms, cardiovascular disease, hypertension, physical disability) and mental health disorders (e.g. depression, generalized anxiety, suicide attempts), and lifestyle habits (e.g. smoking, alcohol use, reduced physical activity) [8,9]. Among older adults, evidence has shown that insomnia is one of the most consistent risk factor for depression [10]. Furthermore, each insomnia symptom subtype increases the risk of developing depression in the future [11].

The aim of this study was to examine associated factors of depression and insomnia in a community-dwelling sample of elderly people in the area of Larissa, Greece. We investigated a number of demographics, socioeconomic, and health-related characteristics of the participants as possible determinants in order to identify independent predictors of depression and insomnia.

## Materials and methods

Study design and participants

A cross-sectional study was performed. The study population consisted of community-dwelling elderly people who were recipients of the services and active members of Open Care Centers for Elderly People (KAPI) of the Municipality of Larissa, whose number amounted to 12,328 people according to the records of the municipality. Larissa is a provincial city located in central Greece, with a population of approximately 200,000. A sample of 250 elderly people was collected from the study population. Stratified random sampling procedure per KAPI with gender matching was used in recruiting samples. Specifically, out of the total five KAPIs, 50 elderly people (25 men and 25 women) were randomly selected from each one. The response rate between KAPIs ranged from 81% to 88%.

The inclusion criteria were as follows: (1) people aged ≥ 60 years, (2) active members of Open Care Centers for Elderly People of the Municipality of Larissa, (3) self-care ability and independent living ability, (4) no history of prior mental disorder or cognitive impairment, and (5) knowledge of the Greek language and ease of communication.

Measures

Data were obtained using a self-reported and structured questionnaire that consisted of the following three sections:

(a) Individual characteristics: Demographic basics (gender, age, marital status, number of children), socioeconomic (living status, educational level, employment status, monthly income), and health-related characteristics (weight, height, chronic diseases) of the elderly people were recorded. Body mass index (BMI) was calculated from self-reported height and weight as weight/(height)2 and categorized according to the World Health Organization classifications: ≤18.4 kg/m^2^ (underweight), 18.5-24.9 kg/m^2^ (normal), 25.0-29.9 kg/m^2^ (overweight), and ≥30 kg/m^2^ (obese).

(b) 15-Item Geriatric Depression Scale (GDS-15): The GDS short form is a 15-item self-report psychometric instrument that has been validated to indicate depression symptoms in geriatric populations. Its items require a “yes” or “no” response. Ten items indicate the presence of depression when answered positively (yes = 1), while the other five items are indicative of depression when answered negatively (no = 1). The total score of the scale ranges from 0 to 15 points. A higher total score is indicative of a higher risk of depression. The Greek version of GDS-15 was used to assess the levels of depression in this study [12]. A cutoff point of 6/7 on the GDS-15 showed optimum receiver operating characteristics for diagnosing depression in older Greek people aged 60 years or above, with a sensitivity of 92% and a specificity of 95%. The scale has shown high internal consistency reliability with a Cronbach’s alpha value of 0.94. In this study, Cronbach’s alpha coefficient was 0.83.

(c) Athens Insomnia Scale (AIS): The AIS is an eight-item self-assessment instrument, originally developed in Greek [13], for quantifying sleep difficulty according to the International Classification of Diseases, 10th Revision, criteria. The first five items pertain to sleep induction, awakenings during the night, final awakening, total sleep duration, and sleep quality. The last three items refer to well-being, functioning capacity, and sleepiness during the day. Each item is rated on a four-point scale ranging from 0 (“no problem at all”) to 3 (“very serious problem”). Hence, the overall AIS score ranges from 0 to 24 points, with higher scores reflecting more severe sleep difficulties. A cutoff point of 5/6 on the AIS was used to establish the diagnosis of insomnia, with 93% sensitivity and 85% specificity. In this study, Cronbach's alpha coefficient for the scale was 0.80, indicating good internal consistency reliability.

Data collection

Data collection was carried out at the five KAPIs of the Municipality of Larissa, during the months of September to December 2019, after obtaining permission from the competent municipality office. The completion of the questionnaires was done through face-to-face interviews by the researcher during the stay of the elderly people in KAPI. Prior to the interview, all participants were given explanations on the purpose of the survey and signed a form of informed consent. The research was executed following the principles of confidentiality, anonymity, and informed consent, as outlined by the Declaration of Helsinki and its subsequent revisions.

Statistical analysis

Descriptive and inferential statistical methods were generated. Continuous variables are presented with mean, standard deviation, and range (min - max), while categorical variables are presented as absolute (n), and relative (%) frequencies. The occurrence of depression and insomnia was used as the outcomes (dependent variables) of the under research correlations, while the demographic, socioeconomic, and health-related characteristics were used as the determinants (independent variables). Odds ratio (OR) with 95% confidence interval (CI) was used as a measure of association. Associations between potential prognostic determinants and outcome were examined using univariate logistic regression analysis. Predictors univariately associated with outcome (p < 0.10) were included in a multivariate logistic regression model (backward method based on maximum likelihood). The fit of the multivariate model was assessed using the Hosmer-Lemeshow goodness-of-fit test. All reported p-values were two-tailed, and a p-value under 0.05 was considered statistically significant. Statistical analysis of the empirical data was performed with the Statistical Package for Social Sciences (SPSS) software, Version 22.0 (IBM Corp., Armonk, NY, USA).

## Results

Sample characteristics

A total of 250 elderly people living in the community participated in this study (125 men and 125 women). The mean age was 74.86 years (SD = 7.90), and the highest percentage of participants was in the age range of 70-79 years (38.8%). Overall, the majority of the respondents were married (54.8%), had one to two children (72.8%), were primary school graduates or had completed some of its classes (54.4%), and were not working (71.2%). According to health-related factors, 66.8% of the elderly people were overweight or obese and 42.0% reported chronic diseases. The detailed demographic, socioeconomic, and health-related characteristics of the sample are illustrated in Table 1.

**Table 1:** Individual characteristics of the participants (n=250) SD, standard deviation

Characteristics	Categories	n	%
Gender	Male	125	50.0
	Female	125	50.0
Age (years)	60–69	74	29.6
	70–79	97	38.8
	≥80	79	31.6
	Mean ± SD	74.86 ± 7.90	
	Range	60–93	
Marital status	Married	137	54.8
	Single	13	5.2
	Divorced	12	4.8
	Widowed	88	35.2
Number of children	0	18	7.2
	1–2	182	72.8
	≥3	50	20.0
Living arrangement	With family	170	68.0
	Alone	80	32.0
Highest level of education	No formal education	45	18.0
	Primary	91	36.4
	Secondary	79	31.6
	Tertiary	35	14.0
Employment status	Working	72	28.8
	Not working	178	71.2
Monthly income (in Euro)	≤500	36	14.4
	501–1000	123	49.2
	˃1000	91	36.4
Body mass index (kg/m^2^)	18.5–24.9 (normal)	83	33.2
	25.0–29.9 (overweight)	119	47.6
	≥30.0 (obese)	48	19.2
	Mean ± SD	26.99 ± 3.42	
	Range	20.7–39.1	
Self-reported chronic diseases	No	145	58.0
	Yes	105	42.0

Prevalence of depression and insomnia in the elderly people

The screening method of the GDS-15 indicated 179 participants without depression (71.6%; 95% CI: 66.1-77.1) and 71 with depression (28.4%; 95% CI: 22.9-33.9). Particularly, 22.0% of the elderly people experienced moderate depression and 6.4% experienced severe depression. According to screening method of the AIS, the prevalence of insomnia for the total sample was 39.2% (95% CI: 33.0-45.4) (Table 2).

**Table 2:** Prevalence of depression and insomnia among elderly people, and the relationship between the GDS score and the AIS score SD, standard deviation; GDS, Geriatric Depression Scale; AIS, Athens Insomnia Scale

Scales	n	%	Correlation
GDS-15 score			r = 0.405
≤6 (absence of depression)	179	71.6	p < 0.001
≥7 (presence of depression)	71	28.4	
7–10 (moderate depression)	55	22.0	
11–15 (severe depression)	16	6.4	
Mean ± SD	4.16 ± 3.56		
Range	0–14		
AIS score			
≤5 (absence of insomnia)	152	60.8	
≥6 (presence of insomnia)	98	39.2	
Mean ± SD	5.84 ± 4.08		
Range	0–20		

The mean score of the elderly people in the GDS-15 was found to be 4.16 (SD = 3.56, ranging from 0 to 14) and in the AIS to be 5.84 (SD = 4.08, ranging from 0 to 20). A positive correlation was observed between the scores of the GDS-15 and those of the AIS (r = 0.405; p < 0.001) (Table 2).

Factors associated with depression

Univariate analyses indicated that elderly people with depression disorder were female, older, currently unmarried, had a lower educational level and monthly income, were overweight or obese, and more likely to suffer from chronic diseases and insomnia. The crude odds ratios are shown in Table 3.

**Table 3:** Results from univariate and multivariate logistic regression analyses for the predictors of depression in the elderly people Hosmer-Lemeshow test p = 0.718; adjusted Nagelkerke R^2^ = 31.6%; overall predictive ability = 77.6%. ^a^Indicates reference category. *p < 0.01. **p < 0.05. ***p < 0.001. COR, crude odds ratio; AOR, adjusted odds ratio; CI, confidence interval

Characteristics	Depression (n = 71)		Non-depression (n = 179)		COR (95% CI)	AOR (95% CI)
	n	%	n	%		
Gender						
Male	24	19.2	101	80.8	1^a^	
Female	47	37.6	78	62.4	2.54 (1.43–4.50)^*^	
Age (years)						
60–69	11	14.9	63	85.1	1^a^	
70–79	27	27.8	70	72.2	2.21 (1.01–4.82)^**^	
≥80	33	41.8	46	58.2	4.11 (1.88–8.97)^***^	
Marital status						
Married	25	18.2	112	81.8	1^a^	1^a^
Not married	46	40.7	67	59.3	3.08 (1.73–5.46)^***^	1.97 (1.01–3.87)^**^
Number of children						
0	3	16.7	15	83.3	1^a^	
1–2	53	29.1	129	70.9	2.05 (0.57–7.39)	
≥3	15	30.0	35	70.0	2.14 (0.54–8.51)	
Living arrangement						
With family	43	25.3	127	74.7	1^a^	
Alone	28	35.0	52	65.0	1.59 (0.90–2.83)	
Highest level of education						
No formal education	22	48.9	23	51.1	1^a^	
Primary	32	35.2	59	64.8	0.57 (0.27–1.17)	
Secondary	13	16.5	66	83.5	0.21 (0.09–0.47)^***^	
Tertiary	4	11.4	31	88.6	0.14 (0.04–0.45)^*^	
Employment status						
Working	16	22.2	56	77.8	1^a^	
Not working	55	30.9	123	69.1	1.57 (0.83–2.97)	
Monthly income (in Euros)						
≤500	23	63.9	13	36.1	1^a^	1^a^
501–1000	40	32.5	83	67.5	0.27 (0.13–0.59)^*^	0.40 (0.17–0.90)^**^
˃1000	8	8.8	83	91.2	0.05 (0.02–0.15)^***^	0.13 (0.04–0.40)^***^
Body mass index (kg/m^2^)						
18.5–24.9 (normal)	13	15.7	70	84.3	1^a^	1^a^
25.0–29.9 (overweight)	38	31.9	81	68.1	2.53 (1.25–5.12)^**^	1.55 (0.70–3.45)
≥30.0 (obese)	20	41.7	28	58.3	3.85 (1.69–8.77)^*^	2.82 (1.13–7.00)^**^
Self-reported chronic diseases						
No	27	18.6	118	81.4	1^a^	
Yes	44	41.9	61	58.1	3.15 (1.78–5.58)^***^	
Insomnia						
No	25	16.4	127	83.6	1^a^	1^a^
Yes	46	46.9	52	53.1	4.49 (2.51–8.06)^***^	3.08 (1.61–5.89)^*^

On multivariate analysis, marital status, monthly income, body weight status, and insomnia were emerged as significant predictors of depression risk (Table 3). Particularly, unmarried (including divorced and widowed) elderly people were two times (95% CI: 1.01-3.87) more likely to have elevated depression symptoms compared to those married. Also, participants with a monthly income of 501 to 1,000 Euros and over 1,000 Euros were 2.5 and 7.7 times, respectively, less likely to be at risk of depression compared to participants with a monthly income of less than 500 Euros. In addition, elderly people classified as obese had 2.8 times (95% CI: 1.13-7.00) more possibility to develop depression disorder than those with normal body weight status. Finally, participants with insomnia were 3.1 times (95% CI: 1.61-5.89) more likely to have depressive symptoms than those without insomnia. This multivariate model explained 31.6% of the variance in depression risk and correctly classified 77.6% of elderly people. According to the Hosmer-Lemeshow test, the data fit the model perfectly (p = 0.718).

Factors associated with insomnia

Results of univariate analyses showed that factors significantly associated with insomnia symptoms in elderly people were gender, age, living arrangement, level of education, employment status, monthly income, body weight status, self-reported chronic diseases, and depression. The crude odds ratios are presented in Table 4.

**Table 4:** Results from univariate and multivariate logistic regression analyses for the predictors of insomnia in the elderly people Hosmer-Lemeshow test p = 0.956; Adjusted Nagelkerke R^2^ = 26.0%; overall predictive ability = 70.8% ^a^Indicates reference category. *p < 0.05. **p < 0.01. ***p < 0.001. COR, crude odds ratio; AOR, adjusted odds ratio; CI, confidence Interval

Characteristics	Insomnia (n = 98)		Non-insomnia (n = 152)		COR (95% CI)	AOR (95% CI)
	n	%	n	%		
Gender						
Male	37	29.6	88	70.4	1^a^	1^a^
Female	61	48.8	64	51.2	2.27 (1.35–3.81)^**^	1.80 (1.01–3.22)^*^
Age (years)						
60–69	17	23.0	57	77.0	1^a^	
70–79	45	46.4	52	53.6	2.90 (1.48–5.69)^**^	
≥80	36	45.6	43	54.4	2.81 (1.40–5.65)^**^	
Marital status						
Married	48	35.0	89	65.0	1^a^	
Not married	50	44.2	63	55.8	1.47 (0.88–2.45)	
Number of children						
0	6	33.3	12	66.7	1^a^	
1–2	72	39.6	110	60.4	1.31 (0.47–3.65)	
≥3	20	40.0	30	60.0	1.33 (0.43–4.13)	
Living arrangement						
With family	58	34.1	112	65.9	1^a^	
Alone	40	50.0	40	50.0	1.93 (1.12–3.32)^*^	
Highest level of education						
No formal education	25	55.6	20	44.4	1^a^	
Primary	43	47.3	48	52.7	0.72 (0.35–1.47)	
Secondary	24	30.4	55	69.6	0.35 (0.16–0.75)^**^	
Tertiary	6	17.1	29	82.9	0.17 (0.06–0.48)^**^	
Employment status						
Working	20	27.8	52	72.2	1^a^	
Not working	78	43.8	100	56.2	2.03 (1.12–3.68)^*^	
Monthly income (in Euro)						
≤ 500	25	69.4	11	30.6	1^a^	1^a^
501–1000	56	45.5	67	54.5	0.37 (0.17–0.81)^*^	0.65 (0.27–1.52)
˃1000	17	18.7	74	81.3	0.10 (0.04–0.25)^***^	0.24 (0.09–0.64)^**^
Body mass index (kg/m^2^)						
18.5–24.9 (normal)	24	28.9	59	71.1	1^a^	
25.0–29.9 (overweight)	52	43.7	67	56.3	1.91 (1.05–3.47)^*^	
≥30.0 (obese)	22	45.8	26	54.2	2.08 (0.99–4.36)	
Self-reported chronic diseases						
No	41	28.3	104	71.7	1^a^	1^a^
Yes	57	54.3	48	45.7	3.01 (1.78–5.10)^***^	2.05 (1.14–3.70)^*^
Depression						
No	52	29.1	127	70.9	1^a^	1^a^
Yes	46	64.8	25	35.2	4.49 (2.51–8.06)^***^	2.41 (1.26–4.60)^**^

These variables were entered into a stepwise logistic regression model. Four factors were included in the final model as significant predictors of insomnia risk: gender, monthly income, self-reported chronic diseases, and depression (Table 4). Specifically, women were 1.8 times (95% CI: 1.01-3.22) at a higher risk of developing insomnia symptoms compared to men. Elderly people with a monthly income of more than 1,000 Euros were 4.2 times less likely to have insomnia than those with a monthly income of less than 500 Euros. Also, participants who reported chronic diseases were 2.1 times (95% CI: 1.14, 3.70) more likely to be at risk of insomnia disorder than those who did not. Elderly people with depression were 2.4 times (95% CI: 1.26-4.60) more likely to suffer from insomnia than those without depression. The final model seems to explain 26.0% in the variation of insomnia risk and can properly classify 70.8% of the studied elderly people. Also, the fit of the multivariate model was perfect (p = 0.956).

## Discussion

The present study was conducted for the purpose of identifying independent predictors of depression and insomnia risk among community-dwelling elderly people in an urban area in central Greece. Several demographic, socioeconomic, and health-related characteristics were examined as potential risk factors. Our findings showed that marital status, monthly income, body weight status, and insomnia were effective on depressive symptoms, which together explained 31.6% of the variance of depression. On the other hand, gender, monthly income, self-reported chronic diseases, and depression were identified as the most important factors in the final model, explaining 26.0% of the variance in insomnia risk.

From the sociodemographic variables in the present study, marital status for depression and gender for insomnia were emerged as significant predictors, confirming previous findings. Particularly, unmarried participants (which in 77.9% of the cases represent widowed individuals) had a two times higher risk of experiencing depressive symptoms than married ones. Several community-based studies demonstrated such association, suggesting that being currently married was a protective factor for depression in elderly people [6,11,14]. In this line, cross-sectional studies based on data from large aging cohort surveys, which included various countries around the world, reported a significantly higher standardized depression prevalence in unmarried (never married/divorced/widowed) than married individuals [5,15]. Depression among elderly people living alone without a spouse might be explained by the fact that unmarried might experience more loneliness and lack of companionship, lower social support, and probably financial hardship and poverty, factors that have been shown to be significant determinants of depression [15,16]. Furthermore, widowhood is an extremely stressful event that changes lifestyle and leads to higher rates of depressive symptoms especially for men than women [5]. It was also found in this study that women were more likely to develop insomnia symptoms than men, with a risk ratio of 1.8. In the same context, previous studies among elderly people conducted in Egypt [17], South Korea [18], and USA [19] showed that females had higher insomnia risk compared to males. Female gender is a strong risk factor for insomnia. According to a meta-analysis of observational studies about the sex differences in insomnia by Zeng et al. [20], females had a significantly higher prevalence of insomnia than males in the general population, with a pooled odds ratio of 1.6. The relationship between female gender and insomnia could be explained by the literature evidence, which shows that females are more likely to be exposed to socioeconomic determinants of insomnia, such as lower educational level and unemployment, physiological (hormonal) factors, and psychological risk factors such as anxiety and stressful life events [21].

Socioeconomic indicators are common determinants of late life depression and insomnia. In the present study, we observed that monthly income was a risk factor for both depression and insomnia, indicating that participants with lower monthly income had a greater likelihood of developing depressive and insomnia symptoms, respectively. Our finding is consistent with several studies and confirms the evidence that depressive [6,16] and insomnia symptoms [19,22] in elderly people are associated with lower socioeconomic status, especially when assessed by indicators of material standards of living. A recent longitudinal study in 7,505 Chinese elderly people by Wang et al. [14] showed a significant correlation between less wealth estimated by household income and depressive symptoms. Also, in a population-based cohort study among elderly people from 17 European countries, individuals classified with depression had a lower household net income [15]. In the study of Ma et al. [23], which included a population of 3,045 older Chinese adults living in the community, participants having no fixed income and less financial support during difficulty were more likely to be at risk of insomnia. This link of depression and insomnia with financial hardship could be explained through some mechanisms, including financial strain, exposure to stressful, unsafe and unstable environments, reduced opportunity of education, low social support, and unhealthy lifestyle behaviors such as smoking, drinking, and obesity [2,8,9]. Another way of looking at this relationship is that less wealth has been associated with a faster decline in age-related functional abilities and phenotypes such as physical capability, sensory function, physiological function, cognitive performance, emotional well-being, and social function [24].

We also found a significant association between body weight status assessed by BMI and occurrence of depression, suggesting that obese elderly people had higher odds of being depressed. Similarly, a longitudinal study conducted by Dearborn et al. [25] among community-dwelling older adults residing in New York demonstrated that both objectively measured and self-reported body mass index were positively associated with concurrent and future depressive symptoms. In another multicenter study, which included baseline data from 17 European countries, the percentage of obesity among depressed and non-depressed elderly people was 29% and 23%, respectively (p < 0.001) [15]. Furthermore, a large community-based study in Southern Brazil by Goes et al. [26] reported that obese elderly people had higher prevalence of being depressed than those with normal weight (OR = 2.34). The relationship between body weight status and depression is multifactorial and seems to be influenced by biological, psychological, and behavior-related factors [27]. Elderly people usually have chronic diseases and health conditions that increase their risk of developing depression. Hence, obesity adversely affects these conditions, resulting in greater disability for the elderly. On the other hand, some studies have shown a negative association between BMI and depressive symptoms [28]. Although the available literature documents the link between body weight status and depression in the elderly, this relationship remains inconsistent.

In this study, the presence of chronic diseases emerged as an important risk factor for developing insomnia disorder. Specifically, participants who reported chronic diseases were more likely to suffer from insomnia symptoms, with a risk ratio of 2.1. Supporting this finding, Ayoub et al. [17] reported that both the presence and number of chronic diseases increase the risk of insomnia in community-dwelling Egyptian elderly people. According to Ma et al. [23] in a large cross-sectional study, the proportion of Chinese elderly people with insomnia measured by AIS was significantly higher among those who reported doctor-diagnosed chronic diseases. In this line, a nationwide epidemiological study in South Korea by Kim et al. [18] found that sleep problems and insomnia among the elderly were closely related to their lifetime history of physical illness. Additionally, in a recent community-based study conducted by Dangol et al. [22] among Nepalese older adults, both the presence of comorbid diseases and the use of regular medicine at present were positively associated with insomnia. In general, the existing body of literature suggests that poor health status in the elderly is significantly associated with a higher likelihood of suffering from insomnia symptoms [19].

Quantitative data suggest the comorbidity model between depression and insomnia in elderly people. In the present study, 39.2% of the participants had insomnia, which was identified as a strong predictor for depressive symptoms, as the insomniac individuals were 3.1 times more likely to be at risk of depression. This finding is in agreement with previous reports that examined the effect of insomnia on depression among older adults [6,11]. A recent meta-analysis of 34 prospective cohort studies by Li et al. [10] showed a significant positive relationship between insomnia and depression, with a pooled relative risk of insomnia to predict depression of 2.3. On the other hand, in our study, 28.4% of the total sample was indicated with depression, which was emerged as a strong predictor for insomnia symptoms, as the depressed elderly people were 2.4 times at a higher risk of developing insomnia. This is an expected result, as the positive effect of depression on sleep disturbances and insomnia has been consistently observed in previous studies conducted in geriatric populations [17,18]. Our findings confirm the epidemiological evidence for the bidirectional predictive relationship between depression and insomnia, as the presence of one increases the risk of developing the other [29,30]. These two health disorders seem to share common neurobiological underpinnings [8].

This epidemiological study had some inherent limitations. First, self-administered psychometric instruments focus on the subjective symptoms, which cannot replace the objective clinical criteria. Second, self-report of health-related characteristics may reduce the internal validity of research due to information bias (recall and social acceptance). Third, data were derived exclusively from members of Open Care Centers for Elderly People and not from the general elderly population. Thus, the use of a single study site diminishes the generalizability of the study findings. Fourth, data were collected in a cross-sectional survey. Consequently, the associated factors should be interpreted with caution, as well as a cross-sectional design cannot establish causality. Despite these limitations, this community-based study confirms most findings reported by other authors, is an important accession to the literature, and provides additional evidence on risk factors for depression and insomnia among elderly people, using stratified random sampling with gender matching and standardized research instruments.

## Conclusions

Depression and insomnia in aging society remain serious public health issues. This study indicated that depression and insomnia in elderly people living in a community are very closely associated with each other. Marital status, monthly income, body weight status, and insomnia were influential factors in the emergence of depressive symptoms among elderly people, and gender, monthly income, self-reported chronic diseases, and depression in the emergence of insomnia. These specific characteristics can be considered as important indicators in evidence-based practice. Knowledge of risk factors for depression and insomnia can enhance efforts to identify high-risk elderly people, strengthen targeted prevention interventions, and improve their quality of life.

## References

[1] (2021). World Population Ageing 2020. https://www.un.org/development/desa/pd/sites/www.un.org.development.desa.pd/files/undesa_pd-2020_world_population_ageing_highlights.pdf.

[2] Sözeri-Varma G (2012). Depression in the elderly: clinical features and risk factors. Aging Dis.

[3] R Assariparambil A, Noronha JA, Kamath A, Adhikari P, Nayak BS, Shankar R, George A (2021). Depression among older adults: a systematic review of South Asian countries. Psychogeriatrics.

[4] Volkert J, Schulz H, Härter M, Wlodarczyk O, Andreas S (2013). The prevalence of mental disorders in older people in Western countries - a meta-analysis. Ageing Res Rev.

[5] Richardson RA, Keyes KM, Medina JT, Calvo E (2020). Sociodemographic inequalities in depression among older adults: cross-sectional evidence from 18 countries. Lancet Psychiatry.

[6] Argyropoulos K, Bartsokas C, Argyropoulou A, Gourzis P, Jelastopulu E (2015). Depressive symptoms in late life in urban and semi-urban areas of South-West Greece: an undetected disorder?. Indian J Psychiatry.

[7] Buysse DJ (2013). Insomnia. JAMA.

[8] Patel D, Steinberg J, Patel P (2018). Insomnia in the elderly: a review. J Clin Sleep Med.

[9] Nguyen V, George T, Brewster GS (2019). Insomnia in older adults. Curr Geriatr Rep.

[10] Li L, Wu C, Gan Y, Qu X, Lu Z (2016). Insomnia and the risk of depression: a meta-analysis of prospective cohort studies. BMC Psychiatry.

[11] Chen TY, Saito Y (2021). Longitudinal effects of nocturnal insomnia symptom subtypes and nonrestorative sleep on the incidence of depression among community-dwelling older adults: results from the Health and Retirement Study. Sleep Med.

[12] Fountoulakis KN, Tsolaki M, Iacovides A, Yesavage J, O'Hara R, Kazis A, Ierodiakonou C (1999). The validation of the short form of the Geriatric Depression Scale (GDS) in Greece. Aging Clin Exp Res.

[13] Soldatos CR, Dikeos DG, Paparrigopoulos TJ (2000). Athens Insomnia Scale: validation of an instrument based on ICD-10 criteria. J Psychosom Res.

[14] Wang Z, Yang H, Guo Z, Liu B, Geng S (2019). Socio-demographic characteristics and co-occurrence of depressive symptoms with chronic diseases among older adults in China: the China longitudinal ageing social survey. BMC Psychiatry.

[15] Horackova K, Kopecek M, Machů V, Kagstrom A, Aarsland D, Motlova LB, Cermakova P (2019). Prevalence of late-life depression and gap in mental health service use across European regions. Eur Psychiatry.

[16] Domènech-Abella J, Mundó J, Leonardi M (2018). The association between socioeconomic status and depression among older adults in Finland, Poland and Spain: a comparative cross-sectional study of distinct measures and pathways. J Affect Disord.

[17] Ayoub AI, Attia M, El Kady HM, Ashour A (2014). Insomnia among community dwelling elderly in Alexandria, Egypt. J Egypt Public Health Assoc.

[18] Kim WH, Kim BS, Kim SK, Chang SM, Lee DW, Cho MJ, Bae JN (2013). Prevalence of insomnia and associated factors in a community sample of elderly individuals in South Korea. Int Psychogeriatr.

[19] Endeshaw YW, Yoo W (2016). Association between social and physical activities and insomnia symptoms among community-dwelling older adults. J Aging Health.

[20] Zeng LN, Zong QQ, Yang Y (2020). Gender difference in the prevalence of insomnia: a meta-analysis of observational studies. Front Psychiatry.

[21] Suh S, Cho N, Zhang J (2018). Sex differences in insomnia: from epidemiology and etiology to intervention. Curr Psychiatry Rep.

[22] Dangol M, Shrestha S, Rai Koirala SK (2019). Insomnia and its associated factors among older people of selected ward of Banepa municipality, Nepal. Nurs Open.

[23] Ma Y, Hu Z, Qin X, Chen R, Zhou Y (2018). Prevalence and socio-economic correlates of insomnia among older people in Anhui, China. Australas J Ageing.

[24] Steptoe A, Zaninotto P (2020). Lower socioeconomic status and the acceleration of aging: an outcome-wide analysis. Proc Natl Acad Sci U S A.

[25] Dearborn PJ, Robbins MA, Elias MF (2018). Challenging the "jolly fat" hypothesis among older adults: high body mass index predicts increases in depressive symptoms over a 5-year period. J Health Psychol.

[26] Goes VF, Wazlawik E, D'Orsi E, González-Chica DA (2017). Severe obesity increases the prevalence but not the incidence of depressive symptoms in the elderly-population-based cohort in Southern Brazil. Int Psychogeriatr.

[27] Milaneschi Y, Simmons WK, van Rossum EFC, Penninx BW (2019). Depression and obesity: evidence of shared biological mechanisms. Mol Psychiatry.

[28] Huang C, Kogure M, Tomata Y (2020). Association of serum adiponectin levels and body mass index with worsening depressive symptoms in elderly individuals: a 10-year longitudinal study. Aging Ment Health.

[29] Bao YP, Han Y, Ma J (2017). Cooccurrence and bidirectional prediction of sleep disturbances and depression in older adults: Meta-analysis and systematic review. Neurosci Biobehav Rev.

[30] Sivertsen B, Salo P, Mykletun A (2012). The bidirectional association between depression and insomnia: the HUNT study. Psychosom Med.

